# Increased Guillain-Barre syndrome admissions in Shiraz, southern Iran

**Published:** 2013

**Authors:** Anahid Safari, Afshin Borhani-Haghighi, Seyed Taghi Heydari, Kamran Bagheri Lankarani

**Affiliations:** 1 Research Center for Traditional Medicine and History of Medicine, Shiraz University of Medical Sciences, Shiraz, Iran; 2 Stem Cell and Transgenic Technology Research Center, Shiraz University of Medical Sciences, Shiraz, Iran; 3 Jahrom University of Medical Sciences, Jahrom, Iran; 4 Health Policy Research Center, Shiraz University of Medical Sciences, Shiraz, Iran

**Keywords:** Guillain-Barre Syndrome, Epidemiology, Influenza A Virus, H1N1 Subtype, Pandemics, Periodicity

## Abstract

**Background:**

Guillain-Barre syndrome (GBS) is an acute immune-mediated peripheral neuropathy usually after an incident. This study was performed to investigate the basic epidemiologic features of GBS in south of Iran.

**Methods:**

We studied consecutive patients with GBS in Nemazi Hospital of Shiraz, southern Iran. Demographic characteristics of the subjects, i.e. age, sex, and length of hospital stay were extracted. Information regarding yearly admissions for the entire hospital was also gathered. The prevalence of GBS per 10,000 hospital admissions was calculated and stratified by sex and year. Yearly prevalence was compared using the odds ratio (OR) and 95% confidence intervals (CI).

**Results:**

From January 2000 to December 2009, 389 (232 males and 157 females) patients with GBS were admitted in our center. The mean age of patients was 29.8 ± 23.0 years and their mean length of hospital stay was 12.9 ± 11.6 days. The overall mortality rate was 6%. The ratio of admissions due to GBS to the total admissions was significantly higher in 2009, the year of an influenza A (H1N1) virus pandemic.

**Conclusion:**

There appears to be an increase in the incidence of GBS in Shiraz. This is in parallel with the increasing trend of some other autoimmune diseases.

## Introduction

Guillain-Barre syndrome (GBS) is an acute immune-mediated peripheral neuropathy disorder characterized by rapidly developing motor, sensory or autonomic manifestations. Up to two-third of cases are preceded by an infection.^1^ The proposed mechanism for GBS is that the immune response evoked by an antecedent infection cross-reacts with peripheral nerve components because of molecular mimicry.^1^ While there are noticeably established causative agents, such as Campylobacter jejuni,^1, 2^ there have been emerging patterns of other possible sources. Although there have been reports of seasonal variation in GBS occurrence, there is no constant seasonal variation in different regions of the world.^1, 3^

We studied the yearly variation of GBS admissions in a large tertiary center in south of Iran from 2000 to 2009.

## Materials and Methods

This was a retrospective chart review study. All the consecutive patients referred from January 1, 2000 to December 31, 2009 to Namazi Hospital, affiliated to Shiraz University of Medical Sciences, Shiraz, Iran, who fulfilled the National Institute of Neurological Disorders and Stroke criteria for GBS^4^ (ICD-9: 357.0) were recruited. This was the main referral center for neurological disorders in Shiraz with a population of 1,455,073 (2009 estimation^5^). Patients with clinical presentations and laboratory profiles more consistent with other acute neuropathies were excluded from the study.

Patients’ demographics including age, sex, and duration of hospital stay were extracted. Information regarding yearly admissions for the entire hospital was also gathered.

### Statistical analysis

GBS frequency per 10,000 hospital admissions was calculated by sex and year. Yearly rates were calculated, and a comparison across groups using odds ratio (OR) with 95% confidence intervals (CI) was made. Statistically significant association was deemed if 95% CI for OR did not include 1.0. To compare the mean of duration of admission and age with year and season, analysis of variance (ANOVA) was used. Independent sample Student's t-test was used for comparing the mean of duration of admission and age with sex and outcome. In a separate analysis the patients were divided into two groups according to age (< 15 versus ≥ 15 years of age). To asses any differences between the two groups regarding year of admission the chi-square analysis was used. The analyses were performed using SPSS software (SPSS Inc., Chicago, IL, USA). All tests for statistical significance were two-tailed, with the level of significance at P < 0.05.

## Results

From January 2000 to December 2009, 389 patients with GBS (232 males and 157 females) were admitted in our center. Mean age of patients was 29.8 ± 23.0 years, mean hospital stay duration was 12.9 ± 11.6 days and overall mortality was 6%. Mean age (Table 1) and hospital stay (Table 2) based on sex, year of admission, and outcome have been provided. Odds ratio was calculated for patients admitted with GBS based on sex and year (Table 3).

**Table 1:** Mean age in years based on sex, year of admission and outcome for patients admitted with Guillain-Barré syndrome

		N	Mean (95% CI)	P
Sex	Male	157	25.9 (22.6-29.3)	0.006
	Female	232	32.5 (29.4-35.5)	
Year	2000	22	28.0 (18.4-37.6)	0.827
	2001	33	24.9 (17.4-32.4)	
	2002	33	26.2 (18.4-34.0)	
	2003	37	27.4 (19.9-34.9)	
	2004	27	29.3 (19.6-38.9)	
	2005	33	30.3 (22.1-38.5)	
	2006	39	34.5 (26.4-42.6)	
	2007	46	31.2 (24.3-38.2)	
	2008	50	30.9 (24.5-37.4)	
	2009	69	31.3 (25.6-37.0)	
Outcome	Discharged	344	28.9 (26.5-31.3)	0.001
	Death	23	45.9 (35.4-56.4)	

**Table 2:** Mean duration of admission in days based on sex, year of admission and outcome for patients admitted with Guillain-Barré syndrome

		N	Mean (95% CI)	P
Sex	Male	156	13.6 (11.8-15.3)	0.331
	Female	232	12.4 (10.9-14.0)	
Year	2000	22	10.6 (4.3-16.9)	0.261
	2001	33	12.1 (9.6-14.6)	
	2002	33	10.5 (8.6-12.4)	
	2003	37	11.7 (7.5-15.9)	
	2004	27	15.3 (10.6-19.9)	
	2005	33	11.4 (9.3-13.5)	
	2006	38	10.0 (7.2-12.8)	
	2007	46	15.2 (11.4-18.9)	
	2008	50	13.8 (10.4-17.2)	
	2009	69	15.0 (11.2-18.7)	
Outcome	Discharged	344	12.6 (11.4-13.8)	0.008
	Death	22	19.5 (11.0-28.1)	

**Table 3:** Odds ratio for patient's admission with Guillain-Barré syndrome based on sex and year of admission

		Total number of hospital admissions	Number of admitted GBS patients	GBS patients per 10,000 admissions	Odds ratio¥ (95% CI)	P
Sex	Male	128611	162	10.0	1	-
	Female	233297	234	12.6	1.18 (0.96-1.44)	0.113
Year	2000	24676	22	8.9	1	-
	2001	28267	33	11.7	1.31 (0.76-2.25)	0.328
	2002	29996	33	11.0	1.23 (0.72-2.12)	0.445
	2003	32155	37	11.5	1.29 (0.76-2.19)	0.344
	2004	34410	27	7.9	0.88 (0.50-1.55)	0.657
	2005	32955	33	10.0	1.12 (0.66-1.93)	0.673
	2006	36112	39	10.8	1.21 (0.72-2.04)	0.472
	2007	37918	46	12.1	1.36 (0.82-2.26)	0.235
	2008	40863	50	12.2	1.37 (0.83-2.27)	0.216
	2009	40338	69	17.1	1.92 (1.19-3.1)	0.008

¥ Odds ratio was created by assuming the number of admitted patients with GBS per 10,000 total admission in 2000 or male sex as reference; GBS = Guillain-Barre syndrome

**Figure 1:**
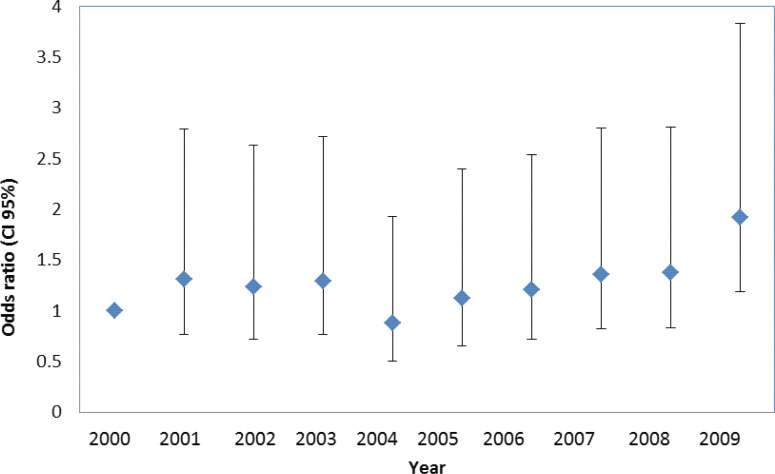
Odds ratio (95% CI) for Guillain-Barre syndrome patients per 10,000 admissions based on year of admission

Mean age was significantly more in females (32.5 ± 23.8 versus 25.9 ± 21.3 years, P = 0.006) and deceased patients (45.9 ± 24.2 versus 28.9 ± 22.7 years, P = 0.001) (Table 1). Duration of hospital stay was significantly more in deceased patients (19.5 ± 19.3 versus 12.6 ± 11.1 days, P = 0.008) (Table 2). Mean age or duration of hospital stay of those admitted for GBS did not differ significantly in the 10 year period (Tables 1 and 2). There was no difference in the duration of admission between the two sexes (for males 12.3 ± 11.6 days and for females 13.7 ± 11.1 days, P = 0.239). Although there was no yearly trend increase in general, calculated odds ratio for acquiring GBS in 2009 was significantly more than any preceding year included in the study. There was no difference between the sexes with regard to this up rise (Table 3, Figure 1). Comparing pediatric patients (less than 15 years, n = 129, 33%) and patients 15 years or older (n = 260, 67%), there was no difference with regard to year of acquiring disease (P = 0.870).

## Discussion

GBS occurs world-wide with an incidence of 1 to 2 per 100,000 per year.^6, 7^ There are only two reports of the epidemiology of the disease in Iran, both confined to the northwestern regions. They estimated the incidence rate at 2.11^8^ to 2.27^9^ per 100,000/year. As the current study was not a population-wide study no incidence rate per year could be calculated, instead we presented the incidence as per 10,000 hospital admission/year.

We could not explain the significantly higher age in our female patients or the significant increase in hospital duration in those deceased.

This study has a number of limitations. Firstly, it is a single center study and may not represent the overall pattern of disease in the region. However, as Nemazi hospital is the largest referral center in the region capable of handling GBS patients, and most cases diagnosed with GBS which are hospitalized,^7^ we found it valid to report our findings. It is speculated that the relative flow of general admissions has been relatively stable over the 10 year period in our center. This is on the grounds that its burden of handling such cases has been substantially large and stable in size over the years included in this study. Secondly, we have to normalize our GBS admissions by the total admissions of the center which includes a variety of reasons for admission.

We also found a significant temporal relationship in the rise of GBS patients with the occurrence of the 2009 pandemic influenza A (H1N1)^10^ (Table 3). The finding of temporal association of GBS and H1N1 infection is just speculative but not evidence based. Although data is scarce, there is a thorough review of currently published literature on the possibility of increased risk of GBS after contact with the influenza virus.^11^ However sophisticated immunological studies to document for a causal relationship are needed. Considering the low number of patients admitted with acute flaccid paralysis, it seems cost beneficial to prospectively collect and freeze a sample of serum from patients admitted with acute flaccid paralysis for future analysis. Data from our latter two suggestions can help immensely in testing the hypothesis that H1N1 can trigger a rise in GBS admissions. Data about epidemiology of GBS in Iran is sparse. A crude annual incidence rate of 2.11/100,000 population has been reported from Northwest of Iran but there is not much data from other regions. Also, there has not been any previous published literature about annual incidence of GBS in Iran.^9^

## Conclusion

There appears to be a rise in the incidence of Guillain-Barre syndrome in Shiraz, Iran. This is in parallel with increasing trend of some other autoimmune disease. Furthermore, there have been some reports of increasing trend of other autoimmune diseases such as multiple sclerosis in Iran.^12^ Meticulous basic and clinical studies about cause of this trend is highly recommended.
